# Coagulase-Negative Staphylococci as an Etiologic Agent of Ovine Mastitis, with a Focus on Subclinical Forms

**DOI:** 10.3390/antibiotics12121661

**Published:** 2023-11-25

**Authors:** Marios Lysitsas, Vassiliki Spyrou, Charalambos Billinis, George Valiakos

**Affiliations:** 1 Faculty of Veterinary Science, University of Thessaly, 43100 Karditsa, Greece; mlysitsas@uth.gr (M.L.); billinis@uth.gr (C.B.); 2 Department of Animal Science, University of Thessaly, 41334 Larissa, Greece; vasilikispyrou@uth.gr

**Keywords:** coagulase-negative Staphylococci, ovine mastitis, subclinical mastitis, PRISMA guidelines, diagnostic procedures, antibiotic resistance

## Abstract

The objective of this review is to investigate the distribution and the characteristics of coagulase-negative Staphylococci (CoNS) implicated in ovine mastitis, and especially in subclinical cases, in order to provide a global perspective of the current research data and analyze specific critical aspects of the issue. PRISMA guidelines were implemented in the search of the last 20 years of the related literature in two databases. In total, 139 studies were included in this review. Relevant data were tracked down, assembled, and compared. Regarding the geographical distribution, most studies originated from Europe (68), followed by South America (33). Lacaune was the most examined breed, while *S. epidermidis* was the predominantly identified species, representing approximately 39% of the obtained isolates. Antibiotic resistance in the relevant bacteria was documented mostly for Penicillin (32.8%) and Amoxicillin (32.1%), while biofilm- and toxin-associated genes were encountered in variable rates because significant inequalities were observed between different articles. Significantly higher rates of antimicrobial resistance were detected in Asia and South America compared to Europe. Finally, the diagnostic procedures carried out in the respective studies were evaluated. Conventional culture and biochemical tests were mostly performed for simple strain identification; therefore, further molecular investigation of isolates should be pursued in future studies, as this will provide important data regarding specific aspects of the implication of CoNS in ovine mastitis.

## 1. Introduction

Mastitis, the inflammation of the mammary gland, is a major trouble in breeding ruminants, including sheep, as it causes significant economic losses, mostly due to a reduction in milk yield, the downfall of its quality, and rejection after antibiotic administration [1,2]. Furthermore, it constitutes a major welfare issue for the infected animals, and it is associated with increased animal replacement and veterinary expenses [3,4]. The subclinical type of the disease requires a more complex and challenging approach because it is commonly spread in flocks, affecting a considerable percentage of the lactating ewes, and it is, in some cases, misdiagnosed [5].

Coagulase-negative Staphylococci (CoNS) are opportunistic pathogens regularly associated with intramammary infection (IMI) in ruminants, and mostly in subclinical cases. During the last years, this group has become a main etiologic agent of ruminant mastitis [6]. Furthermore, recent studies indicate that these species are capable of causing more severe tissue damage in the mammary gland than was previously considered [7]. The bacterial species most frequently identified in cases of mastitis in sheep are *S. epidermidis*, *S. chromogenes*, *S. simulans*, *S. xylosus*, and *S. haemolyticus* [2,3,8].

Several studies have been performed worldwide investigating the prevalence, etiology, predisposing factors, and pathogenesis of ovine mastitis, as well as the implication of Staphylococci in the development of the disease.

The objective of this review is to collect and analyze data regarding the implication of CoNS in ovine mastitis, with a focus on the more challenging, subclinical type of the disease. A global perspective of the subject will be presented, and recently obtained information will be provided, by including studies published worldwide during the last two decades (2003–2023). Moreover, critical aspects of the subject will be investigated, such as pathogens’ prevalence, virulence, antibiotic resistance, and public health concerns.

## 2. Results

### 2.1. Geographical Distribution

The distribution of the included articles in relation to the continent of origin is:

- Europe: 67
- South America: 33
- Asia: 21
- Africa: 12
- North America: 5
- Europe and South America: 1

Assuming equal proportions, significantly more studies originate from Europe, while significantly fewer studies originate from Africa and North America (Chi-square test: 88.21; Degree of freedom: 4; *p*-value: <0.001).

**Figure 1:**
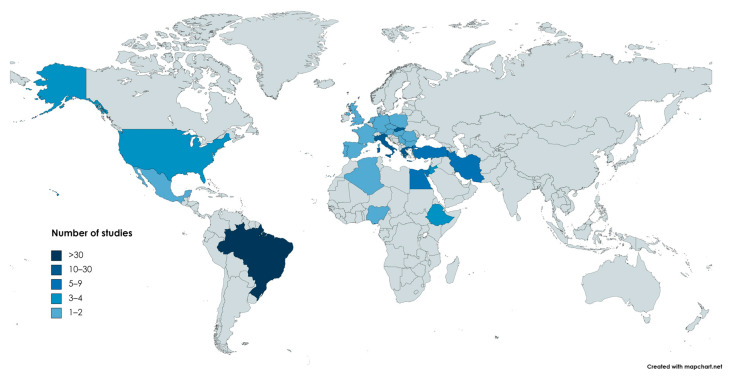
Distribution of the studies (countries in shades of blue) included in this review throughout the world. Color indicates the arithmetical range to which the number of relevant articles from the corresponding country belongs. (https://www.mapchart.net/world.html, accessed οn 20 September 2023).

In reference to the country/area of origin, the respective distribution is presented in Table 1 and visualized in the map presented in Figure 1.

### 2.2. Relevant Findings of the Studies per Country

Due to the number of studies and the size of the data, only generic information is presented in this part of the results section. More detailed data about the findings of the selected articles are available in the following parts and the respective tables. The order of Table 2 is used to present the findings per country.

#### 2.2.1. Brazil

Thirty-three studies from Brazil were selected. They were published from 2009 to 2022. Over 12,000 milk samples were totally tested in all of them.

Intramammary infections in Santa Ines ewes were investigated in several studies [9,10,11,12,13,14,15,16,17,18,19,20,21]. Both Santa Ines and Morada Nova breed ewes were researched in four other articles [22,23,24,25], while Santa Ines and Bergamacia were researched in one [26]. Samples were received from farms located in various regions, such as Sao Paolo, Pernambuco, Bauru, Para, Montes Claros, Sergipe State, and Parana State.

Lacaune and their crossbreeds were also examined in seven studies [27,28,29,30,31,32,33], which were carried out in Chapeco-SC, Rio Grande do Sul, Santa Catarina, and Minas Gerais. Coagulase-negative Staphylococci were regularly obtained, especially from subclinical udder infections. Ovine milk samples of various breeds were tested in a few more articles, such as Coriedalle and Texel [34], Santa Ines, Ile de France, Dorper, and Texel [35,36,37]. Finally, ovine Staphylococci and their phenotypic and molecular characteristics were investigated in four more studies [38,39,40,41]. In some cases, concerning results regarding drug resistance were reported [39].

#### 2.2.2. Greece

A total of 18 studies from Greece are included in this review. They were published from 2007 to 2022. The majority of the samples were received from central and northern parts of the country. Regarding breeds, Chios (5), Karagouniko (4), and Lacaune (4) were the most frequently encountered.

In particular, two studies that investigated aspects of milking and mammary glands’ health were accomplished for ewes of the Karagouniko breed [42,43]. Control measures for subclinical mastitis [44], the survival of CoNS species during the dry period [45], and the effect of the drying-off procedure [46], were investigated in Chios ewes in three research studies carried out in northern and central Greece. The effects of the drying-off procedure in the mammary health status were also examined in Lacaune-cross sheep in Central Greece [47]. In all of these studies, CoNS were regularly obtained, identified, and, in some cases, molecularly characterized [45,46]. Furthermore, the hypothesis that parasitic infections could predispose ewes to ovine subclinical mastitis was investigated in two articles [48,49]. The potential of specific enzymes for the diagnosis of subclinical mastitis in small ruminants was examined in another study [50]. The consequences of reduced vitamin A administration in the health of the mammary gland of Mytilene breed ewes were investigated [51], while *mph*C-positive Staphylococci obtained from cases of ovine subclinical mastitis were described in another article [52]. Extensive countrywide research on subclinical mastitis was accomplished, while various factors, such as breeds, susceptibility of the isolates, including numerous CoNS, and biofilm production, were investigated [53,54,55,56]. The efficacy of a vaccine against staphylococcal mastitis was evaluated in a study carried out in central Greece [57], while the association of subclinical mastitis with biotic and abiotic factors was examined for sheep of the Sfakia breed in Crete [58]. In these studies, a total of 272 and 652 CoNS strains were isolated, respectively. Finally, 33 *S. epidermidis* strains obtained in the two previous studies [53,57] were submitted for multi-locus sequence typing (MLST) and evaluation of their susceptibility profiles [59].

#### 2.2.3. Italy

A total of 16 studies from Italy was selected for this review. They were published from 2005 to 2022. A large number of examined milk samples was included in most of them (total > 60,000 samples), while the isolated bacteria were, in various cases, submitted to molecular assays [60,61,62,63,64,65,66,67].

Several studies originated from Sardinia. The antibiotic residues in ovine milk samples were evaluated [68], a clinical investigation of mastitis cases in 2198 Sarda sheep was carried out [60], and 226 CoNS strains of ovine milk origin were identified through PCR-Restriction Fragment Length Polymorphism (RFLP) assay [61], while 131 *S. epidermidis* isolates from the previous study were submitted for susceptibility testing and genotyping [62]. Furthermore, the effects of infection in Somatic Cell Count (SCC) and milk yield of Sarda-breed ewes were examined [69], enterotoxigenic and methicillin-resistant CoNS isolates from ovine milk were identified and molecularly characterized [63], and the efficacy of an essential oil in teat disinfection was evaluated [70]. Finally, a total of 199 *S. epidermidis* strains isolated from sheep milk were investigated for biofilm production, quorum sensing system, and virulence factors [64]; pathogens associated with small ruminant mastitis were identified using a Matrix-Assisted Laser Desorption/Ionization Time-of-Flight Mass Spectrometer (MALDI-TOF MS) and PCR-RFLP [66], and a comparative profiling of 70 human and 125 ovine Staphylococci was accomplished [67].

Regarding other regions, an investigation of the associations among genetic parameters, somatic cell score, and mammary infection status was carried out in Valle del Belice ewes [71]. For the same breed, an assessment of the genetic background of pathogen-specific mastitis resistance was accomplished [72]. A survey of small ruminant mastitis during 2013–2014 included data from 23,040 ovine samples obtained in different areas of the country [73]. In Sicily, the efficacy of intramammary-infused *Lactococcus lactis* against staphylococcal mastitis was examined in 67 ewes [74], while in the Piedmont region, microbial agents from macroscopically healthy mammary glands of small ruminants were obtained and examined [75]. Furthermore, 73 CoNS isolates from ovine milk were submitted to a phenotypic and molecular investigation [65].

#### 2.2.4. Slovakia

Twelve articles from Slovakia were included, which were published from 2009 to 2022. Local breeds were mostly studied, such as Tsigai and Valaska. Furthermore, the region of the sampling (when available) was mainly located in the eastern parts of the country.

Specifically, 240 CoNS isolates from ovine milk were identified and genotyped [76], whereas in another article, the production of enterotoxins in Staphylococci of the same origin was evaluated [77]. The prevalence and characteristics of mastitis pathogens in sheep farms located in marginal parts of the country were examined in another study. Among these pathogens, 76 CoNS isolates were identified [78]. The antibiotic resistance profiles of 288 and 158 CoNS isolates obtained from sheep milk during 2015–2017 and 2017–2019, respectively, were investigated in two other articles [79,80]. Susceptibility patterns of 131 common udder pathogens isolated in 2017 and 2018 were examined, and relatively high rates were documented for some agents [81], while the distribution of leukocytes and epithelial cells in Tsigai-breed ewes was researched in association with their udder health status, including cases of CoNS-caused mastitis [82]. In another study, fatty acid profiles of infected ovine milk samples were examined [83]. Moreover, bacterial pathogens and somatic cells in 303 ewes were investigated in 2019 [84], the presence of pathogens in association with SCC and subpopulations of leukocytes in 45 Lacaune ewes were researched [85], and the effect of udder infection on oxidative status was evaluated in 981 ovine milk samples of various breeds [86]. In all of these three studies, CoNS were the prevalent pathogens detected. Finally, in a study accomplished in eastern Slovakia, 44 CoNS strains were obtained from ovine subclinical mastitis cases and submitted to phenotypic and molecular investigation for antibiotic resistance, virulence factors, and biofilm formation [87].

#### 2.2.5. Iran

Eight studies from Iran were selected. They were published from 2003 to 2018 and originated from various regions of the country. Native breeds were mostly researched.

Frequency, causative agents, and enzymatic activity of subclinical mastitis were investigated in 178 ewes in Urmia Province [88], while in the Shahrekord region, two studies were performed on susceptibility and phenotypic characteristics of isolates from 400 and 600 milk samples of native-breed sheep, respectively [89,90]. Additionally, the prevalence and aetiology of subclinical mastitis were researched in ewes from Tabriz [91], Nagadeh [92], and Semnan [93]. In 196 Sangsari-breed sheep from the latter region, the activity of specific enzymes was evaluated as a diagnostic tool for subclinical mastitis [94]. Coagulase-negative Staphylococci were regularly obtained in all of these studies, and they were the prevalent isolated bacteria in several cases [88,91,93,94]. Finally, in another study carried out in West Azerbaijan Province, 27 Staphylococci obtained from cases of ovine subclinical mastitis were phenotypically and molecularly investigated, and high resistance rates were detected for specific agents [95].

#### 2.2.6. Egypt

Six of the included articles originated from Egypt. They were published from 2005 to 2019. Ovine subclinical mastitis was investigated in dairy ewes for two studies carried out in Fayoum [96] and Kafr-el-Sheikh [97] Governorates, with 38 and 45 CoNS isolations, respectively. In the latter article, concerning rates of resistance were documented for some antibiotics [97]. Nine CoNS isolates were also identified from sheep with subclinical mastitis both in the Assiut Governorate [98] and Sharkia Governorate [99]. The susceptibility of udder pathogens, including eight CoNS strains of ovine origin, to antibiotics, essential oils, honey, and plant extracts was evaluated [100]. Finally, the role of Staphylococci in subclinical mastitis was examined in 455 sheep milk samples, with 18 isolates being coagulase-negative [101].

#### 2.2.7. Turkey

Six studies from Turkey were selected. They were published from 2009 to 2019. Three of them included Awassi-breed sheep from the Hatay region. Coagulase-negative Staphylococci were the predominant cause of subclinical mastitis in the first [102], while 70 of the obtained isolates were examined in a second study for phenotypic and molecular characteristics, and relatively high rates were documented regarding resistance to specific antibiotics and biofilm production ability [103]. In the third study, the E-test was evaluated for susceptibility determination of 50 ovine Staphylococci isolates to specific antibiotics [104]. In two other articles, CoNS from milk infections in the Kirikalle region were examined for the identification of susceptibility profiles and specific virulence factors [105,106]. Finally, 31 CoNS isolates from subclinical cases of udder infection in Pirlak sheep were molecularly investigated for specific resistance and toxin-encoding genes [107].

#### 2.2.8. USA

The impact of dry treatment and teat sanitation on the health status of the mammary gland was examined in sheep in Wisconsin. During the research, 73 CoNS strains were obtained from milk samples [108]. The prevalence and aetiology of subclinical mastitis at weaning were investigated in ewes in Wyoming, and four CoNS were isolated [109], while in a study on extensively managed sheep of various breeds in Montana and Idaho, 25 more isolates were identified [110]. In another study, subclinical mastitis was investigated in 42 ewes in Wyoming, and CoNS were the aetiologic agent in 59% of the cases [111].

#### 2.2.9. Ethiopia

Three studies from Ethiopia were included in this review. A total of 531 milk samples were tested, and CoNS were the prevalent isolated pathogens in all cases. Local breeds were examined in farms located in Kafta Humera [112], Haramaya [113], and Jimma [114].

#### 2.2.10. Israel

Three studies with Assaf-breed sheep were included. In the first, subclinical mastitis and changes in milk composition were examined, and 36 CoNS were totally isolated [115]. In the second, dry-off treatment was evaluated in 159 ewes, and, in total, 134 CoNS were obtained [116]. In the third, the effects of udder infection caused by CoNS were investigated in 61 ovine milk samples [117].

#### 2.2.11. Jordan

In all three selected studies from Jordan, Awassi-breed sheep were examined. In total, 1646 ovine milk samples were included, and CoNS along with *S. aureus* were the pathogens mostly detected. These studies were accomplished in southern Jordan [118], northern Jordan [119], and in the Al Balqa region [120].

#### 2.2.12. Other Countries

Two studies were carried out in Algeria with Ouled-Djellal sheep. Antibiotic residues were examined in ovine milk samples [121], while the aetiology of subclinical mastitis was investigated [122]. Sixteen and thirty-four CoNS were isolated, respectively. In Austria, CoNS were the predominantly obtained pathogens in two studies. In particular, 267 [123] and 908 [124] ovine milk samples were examined. Regarding Bulgaria, two studies were caried out by Stoimenov et al. investigating udder pathogens in lactating sheep. In both cases, CoNS were regularly identified [125,126].

Staphylococci from the milk of ruminants were examined in a study in the Czech Republic [127], while methicillin-resistant Staphylococci, including coagulase-negative ones, were identified in another one from samples originating from both the Czech Republic and Slovakia [128]. In France, two studies were carried out, in which 4880 ovine milk samples were tested. Somatic cell count thresholds were investigated [129], while an SCC-based selection for mastitis resistance in sheep was evaluated [130]. Coagulase-negative Staphylococci were the most frequently isolated bacteria in both cases. In an article from Germany, the diagnostic value of CMT in dairy ewes was examined, and during the process, 24 CoNS were detected [131]. Staphylococci of ovine origin were identified through MALDI-TOF MS in Hungary [132], and the prevalence and aetiologic agents of subclinical mastitis were researched in ewes from the Pajacuaran Michoacan municipality, Mexico [133]. Ovine mastitis was also investigated in Bauchi State, Nigeria, and two CoNS strains were identified [134]. In a study of Awassi-breed ewes in Palestine, CoNS were the predominant udder pathogens [135].

In Poland, 108 Staphylococci were obtained from ovine milk samples of native breeds [136]. In a study from Romania, one *S. epidermidis* strain was obtained from a subclinical udder infection [137], and during research carried out in Portugal and Brazil, 20 CoNS isolates were detected [138]. Queiroga investigated sheep mastitis in Alentejo, Portugal, and 249 CoNS were totally isolated from subclinical cases [139], while Queiroga et al. submitted 109 ovine *S. epidermidis* isolates for phenotypic biofilm production and adhesion assays [140]. In Serbia, 25 cases of CoNS subclinical infection were detected [141], while in Spain, the effects of dry-therapy were investigated in lactating ewes [142], and the major udder pathogens were identified in small ruminants [143]. In all of these articles, numerous CoNS were isolated. Texel-breed sheep were examined in two studies accomplished in the Netherlands, in which CoNS were prevalent among the isolated pathogens [144,145]. Finally, in the UK, ovine milk samples were bacteriologically tested in two studies, and in both of them, several CoNS were associated with cases of infection [146,147].

### 2.3. Breeds of Sheep Included in the Studies

Sheep of various breeds are included in the selected articles. Those breeds with more than five references are listed in Table 2.

Studies including Lacaune sheep are distributed worldwide, whereas most other breeds are, unsurprisingly, strongly related to articles from specific countries or regions (Santa Ines—Brazil, Valachian and Tsigai—Slovakia, Chios—Greece, Sarda—Italy, etc.).

### 2.4. Prevalence of Pathogens

In 118 studies, the prevalent pathogen from all of the isolated bacteria is defined; the pathogen was identified in most of the samples investigated, even though, in all studies included in this review, a number of CoNS was isolated. Respective data are presented in Table 3.

In the majority of the studies (95/118, 80.5%) included in this review, cases of mastitis (mainly subclinical) were mostly associated with infections by CoNS, which are the prevalent and main cause of the problem.

### 2.5. Species of Identified CoNS

As presented in Table 4, there were references to several CoNS species from the cases of ovine mastitis. Data from 77 studies, in which the numbers of these species were available, have been included.

As it is clearly noticed, *S. epidermidis* is the predominant species of CoNS in the included studies, as it was observed in approximately 40.0% of CoNS identification cases. This species was detected, at least once, in 61/77 (79.2%) studies, which provided the respective data, while in 34 of them (44.2%), it was prevalent among CoNS. Other species regularly obtained are *S. chromogenes, S. simulans,* and *S. xylosus*. These four species represent over 70% of total cases. Numerous other species are only occasionally obtained.

### 2.6. Antibiotic Resistance

In various studies, antibiotic susceptibility testing has been accomplished. Respective data have been accumulated and presented in Table 5.

The isolates were usually susceptible to the majority of the antibiotics tested. However, relatively higher resistance rates were documented for Penicillin (32.8%), Amoxicillin (32.1%), Ampicillin (28%), Tetracycline (18%), Streptomycin (17.9%), Neomycin (14.8%), and Erythromycin (11.3%).

Because a sufficient number of studies was carried out in Europe, South America, and Asia, the resistance data included in Table 5 are separated according to their origin in Table 6. This task was accomplished in order to identify possible geographical variations in resistance rates.

The highest resistance rates for Ampicillin and Penicillin were documented in Asia (41.05% and 50.85%) compared to South America (32.76% and 31.33%) and Europe (22.31% and 23.23%). Furthermore, oxacillin-resistant strains were detected significantly more frequently in South America than in Europe. Finally, rates against Tetracycline and Erythromycin were also relatively higher in South America (Brazil), while insignificant variations were observed for Cefoxitin, Enrofloxacin, and Gentamicin.

### 2.7. Biofilm Production and Biofilm- and Toxin-Associated Genes

An issue frequently investigated in the selected studies was the ability of the strains to produce biofilm and/or specific toxins, because these factors affect the severity and the persistence of the disease. Relevant available data are accumulated in Table 7.

The ability of CoNS isolates to produce biofilm was detected in higher rates when the plate adhesion technique was carried out (28.7%) compared to Congo Red Agar assay (8.4%). Biofilm-related genes were present in variable percentages of the isolates, with *embp* (78.3%) and *bhp* (31%) the most frequently encountered. A noteworthy fact is that in several cases, a significant inequality existed between the results of the phenotypic and molecular assays, indicating that the presence of the relevant genes is not always interrelated with a respective phenotype.

Toxin genes were rarely identified in the majority of the studies, and in only four articles were relatively higher percentages of positive isolates observed [21,25,26,106]. Three of them originated from Brazil.

### 2.8. Procedures of Microbiological Examination in the Selected Studies

Data regarding the diagnostic procedures followed by each study during the microbiological examination of the ovine milk samples were evaluated. Accumulated data are presented in Table 8.

In the majority of the studies, milk samples were inoculated in blood agar and identified through conventional phenotypic and biochemical tests. MacConkey agar and Mannitol salt agar were the most commonly used selective media during the initial inoculation process. From the available commercial identification kits, API Staph was the most widely used, while in a relatively small number of studies, identification was accomplished through MALDI-TOF MS or VITEK II. Antibiotic susceptibility was assessed mainly through the disc diffusion method.

Data regarding molecular investigation of the CoNS isolates are available in 37 articles. Techniques associated with molecular identification of the species level of the isolates were used in 20 cases. Various molecular assays were carried out occasionally, such as Pulse Field Gel Electrophoresis (PFGE) [45,46,60,62,76], PCR-RFLP [28,60,61,65,66,95], etc. PCR for the detection of ARGs and biofilm-related genes was accomplished in 19 [21,26,27,39,40,41,52,56,59,62,63,65,87,103,105,106,107,138,143] and 10 studies [25,26,27,39,55,64,65,67,103,138], respectively, while in 9 cases, genes associated with the production of enterotoxins were investigated [21,25,26,63,64,67,77,106,107]. The most commonly examined gene was *mec*A, with 16 relevant studies [21,26,27,39,40,41,59,62,63,65,87,103,105,106,107,138]. An interesting fact is that all of the aforementioned articles have been published since 2009, and the majority of them (22/36) were published during the last decade (2014–2023).

## 3. Discussion

Τhe results of this review indicate the importance of CoNS in ovine mastitis, and especially in subclinical cases. In a great number of the selected articles, these species were obtained repeatedly during the examination of ovine milk samples, regardless of breed, country, or culture media.

Countries traditionally associated with small ruminant breeding, such as Brazil, Greece, Italy, etc., accommodate numerous respective studies, including mainly local breeds. The majority of these breeds are dairy; however, research on wool and meat-producing sheep has been accomplished, too. This is anticipated because mastitis is a factor in deficient welfare, increased mortality, and reduced lamb growth [145].

*Staphylococcus epidermidis* was the predominant species identified (Table 4). This is in accordance with previous reports for ovine IMI [3], while *S. chromogenes*, *S. xylosus*, and *S. simulans* are also commonly detected. Moreover, because current knowledge suggests that *S. epidermidis* is a human-adapted species [8], the contribution of human sources to its distribution as well as its zoonotic potential are concerning facts that need further investigation.

Nevertheless, the diagnostic procedures that were carried out in the selected articles could affect the respective results. As it is clearly presented in Table 8, the identification of the pathogen causing udder infection is mostly based on aerobic culture after inoculation of a milk quantity in general purpose media. However, because some microorganisms are fastidious or require specific techniques to be detected, the prevalence of CoNS could be overestimated. When culture-independent methods for the detection of the etiologic agent were carried out, a greater diversity of identified pathogens was observed [8]. Therefore, the modification of the diagnostic procedures in the future could contribute to an alteration of the CoNS predominance in IMI cases.

In reference to antibiotic resistance, the highest rate was observed for Penicillin (unsurprisingly, similar rates were detected for Amoxicillin and Ampicillin) both worldwide and per continent (Table 5 and Table 6). These results correspond to previously published data [2,148]. Tetracycline and Erythromycin were also agents with noteworthy percentages of resistant isolates. Furthermore, comparable rates have been identified for CoNS originating from bovine mastitis [148], while the distribution of ARGs associated with respective phenotypes has been investigated and identified at various occasions in sheep [2,52,56,59,65,103,138]. On the other hand, antibiotics like Fluoroquinolones, Sulphamethoxazole–Trimethoprim, Gentamicin, Cephalosporins, Rifampicin, Vancomycin, and Chloramphenicol exhibited in vitro effectiveness against the grand majority of the relevant strains. It is important to note that the disc diffusion method is the one mainly used in most studies; MIC calculation is something that should be applied on a larger scale in future studies, as it will offer more information on antimicrobial resistance patterns.

Higher resistance rates are detected in Asia and South America compared to Europe (Table 6). This fact is anticipated, because both antibiotic administration and resistance rates are increasing in livestock animals in these areas [149]. Therefore, reduction in the usage of antibacterial agents and preventive measures for the distribution of resistant strains are required. Furthermore, surveillance measures should be established, because recent research suggests that CoNS could act as a reservoir for several transferrable ARGs [150]; therefore, the danger of horizontal or vertical gene transfer at the farm level is not negligible.

Coagulase-negative Staphylococci isolates with an ability to produce biofilm were occasionally encountered using the Congo Red Agar (CRA) method (8.4%), while more bacteria exhibited a positive phenotype when tested using the plate adhesion technique (Table 7). The latter assay is referenced as more sensitive [151,152]; thus, more biofilm-producing bacteria are usually detected. In addition, most associated genes were identified at relatively higher rates (from 9.8% to 78.3%), and this could indicate that a percentage of them could be misdiagnosed by phenotypic tests. However, due to the complexity of the biofilm formation process and the possible implication of more genes that are not comprehensively researched [55], this evaluation constitutes a challenging task.

Finally, toxin-related genes were regularly present in low percentages in the isolates, with some exceptions (Table 7). Staphylococcal enterotoxins (SEs) and toxic shock syndrome toxin (TSST) could contribute to bacterial virulence [3,67]. They were generally correlated with *S. aureus*. Nevertheless, references to enterotoxin-producing CoNS are continuously increasing [153]. This could be concerning given their presence in milk and, consequently, food products, because cases of subclinical mastitis could be misdiagnosed. Therefore, future studies should include a more extensive investigation of the obtained bacteria, including tests for the detection of toxins or the respective genes and the determination of the factors affecting toxin production in cases of mastitis. Consequently, appropriate surveillance measures should be established in order to ascertain the safety of milk and dairy products.

Subclinical mastitis is undoubtedly a troublesome disease in sheep. It is a cause of significant animal welfare issues and financial losses at the farm level. Furthermore, concerns regarding public health arise. The distribution of bacteria or genetic elements associated with antibiotic resistance and specific virulence factors, such as biofilm and toxin production, is possible not only inside the farm but also in relation to its environment through animal transportation, milk, or bio-waste. The prevalence of CoNS in cases of subclinical mastitis is also an important aspect, because they are bacteria with a zoonotic potential [154]. Additionally, even though their implication in food poisoning has not been thoroughly investigated yet, enterotoxin production from relevant strains has been identified [153]. Therefore, surveillance and management measures are essential at the farm level in order to limit the number of cases, achieve an early diagnosis and treatment of infected animals, and prevent further distribution of strains with a pathogenic potential. Novel techniques that could allow for the early detection of mastitis cases, such as the detection of specific biomarkers [2] or infrared thermography [155], could constitute effective tools for veterinarians in this endeavour. Furthermore, the application of molecular techniques, like whole genome sequencing, next-generation sequencing, and MLST technologies, should be further increased in future studies, as they could contribute to a comprehensive investigation of the genetic basis of significant pathogens’ characteristics, like virulence and antibiotic resistance, as numerous respective studies have been accomplished for human isolates [156].

## 4. Materials and Methods

**Figure 2:**
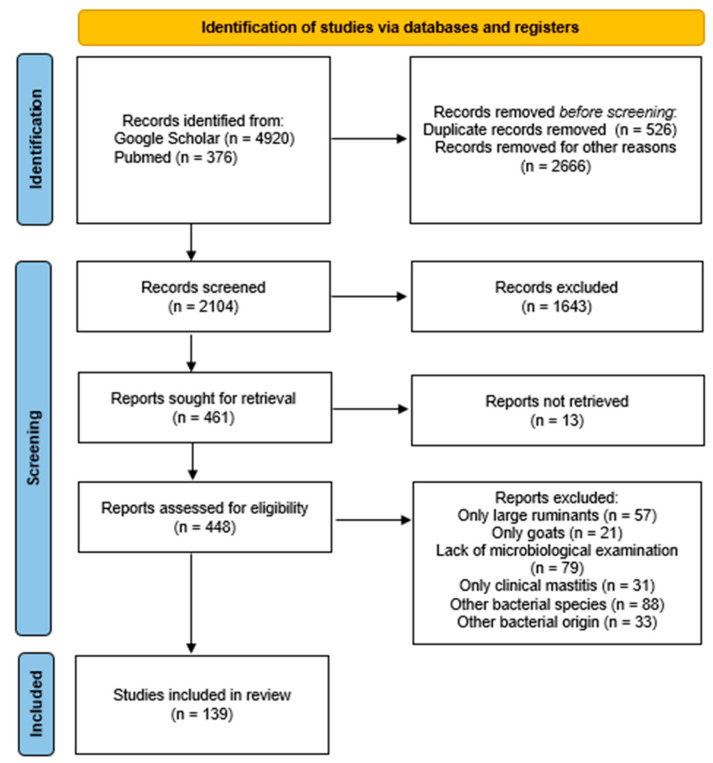
Identification of studies regarding CoNS and ovine mastitis via databases using the PRISMA guidelines [157].

The Preferred Reporting Items for Systematic Reviews (PRISMA) guidelines were implemented for this study [157]. Each individual step of the process is presented in Figure 2. Initially, a search for reviews on the subject of mastitis in sheep was accomplished using the following databases: Google Scholar and PubMed. Only studies published during the last 20 years (2003–2023) were included. In these databases, 5296 studies were found using the keywords coagulase negative, *Staphylococcus*, and ovine mastitis, as well as extra keywords, such as subclinical, milk, and sheep, in various combinations.

All of the selected studies were published in peer-reviewed journals, websites of organizations, books, and dissertations, and they were mostly written in the English language, with a limited number of them published in Portuguese. The initial step was a screening based on the titles of the articles. Articles not related were excluded, like duplicates, studies referring to human medicine, studies referring to other animal species, such as large ruminants and goats, and studies referring to other bacterial species or to CoNS of other origin, except mastitis. Subsequently, the second selection phase was carried out, where the abstracts of the reviewed studies were examined independently and in detail in order to identify their relevance. During this step, studies exclusively including cases of clinical mastitis were excluded due to the focus of this review on the subclinical type. Generic information was collected from each article, such as the author, year of publication, country where the study was conducted, study design, number of samples tested, number of isolates obtained, diagnostic procedures, antibiotic resistance, and virulence of the strains.

In particular, a total of 5296 manuscripts were detected, with 4920 from Google Scholar and 376 from PubMed. A total of 3192 publications were first excluded as their title was completely irrelevant or they were duplicates. Subsequently, the abstracts of the 2104 remaining articles were examined. During this phase, 1643 were rejected because their abstracts were not relevant to the scope of this review, according to the previously referenced criteria. Therefore, 461 studies were left to be examined, and 13 of them could not be retrieved. Among the remaining 448 articles, 57 were rejected as they only concerned large ruminants, 21 were rejected as they only concerned goats, 79 were rejected as they did not include a detailed microbiological examination of the milk samples, 31 were rejected as they only included cases of clinical mastitis, 88 were rejected as they only concerned other bacterial species, and 33 were rejected as the bacteria did not originate from mastitis (bulk milk, environmental samples, etc.). Finally, 139 manuscripts were included in this review.

The generic information extracted from each selected article is presented in Table 9. The country/area of isolation, the breed of sheep, the number of milk samples tested, the number of isolated CoNS, and the prevalent isolated pathogen in the study (the pathogen that was identified in most of the samples investigated, even though in all studies presented here a number of CoNS was isolated) are listed.

## 5. Conclusions

Coagulase-negative Staphylococci are predominant etiologic agents of subclinical mastitis in sheep causing significant economic losses and animal welfare issues. Their distribution in respective cases is worldwide and breed independent. Thus, reduction of their presence at the farm level should be considered of major importance. To achieve this, various actions must take place. Good milking hygiene practices (with proper operation of the milking machines), implementation of cleaning and disinfection programs while keeping the animal’s living environment dry and comfortable, segregation and killing of persistently infected animals, preventive use of antibiotics in the dry period, and, potentially, vaccination are major actions that can be applied. *Staphylococcus epidermidis* is the most frequently encountered species, and its zoonotic potential raises concerns regarding cross-contamination between humans and animals. This highlights the need for further investigation of mastitis in a broader context and with a One Health perspective. Approximately three out of ten strains isolated are Penicillin resistant, while noteworthy rates have also been observed for Tetracycline and Erythromycin, which are concerning facts for the efficacy of these broadly used antibiotics in therapeutic protocols. The dispersion of ARGs through epidemic strains or transferrable genes is possible, especially in regions where higher rates are detected, like Asia and South America; therefore, surveillance measures should be established. Biofilm- and toxin-associated genes have been identified in several articles, with considerable variations observed among the respective rates. The aforementioned results could be affected by the diagnostic approach in the selected studies, because aerobic culture and conventional identification tests are mainly carried out. Thus, the application of more advanced, novel techniques in the future, which could detect cases at an early phase, and further investigation of the isolated strains in a timely manner (especially molecular typing and sequencing technologies), could provide a comprehensive examination of significant, specific aspects of the disease, like strains’ virulence or antimicrobial resistance profiles. Relevant data could contribute to appropriate management and counteracting the consequences of mastitis for animal welfare, economics, and public health.

## Figures and Tables

**Table 1:** Number of studies included in this review per country of origin.

Country	Number of Studies
Brazil	33
Greece	18
Italy	16
Slovakia	12
Iran	8
Egypt, Turkey	6
USA	4
Ethiopia, Israel, Jordan	3
Algeria, Austria, Bulgaria, France, Portugal, Spain, The Netherlands, UK	2
Czech Republic, Czech Republic and Slovakia, Germany, Hungary, Mexico, Nigeria, Palestine, Poland, Portugal and Brazil, Romania, Serbia	1

**Table 2:** Breeds of sheep mostly included in the relevant studies.

Breed	Number of Studies
Assaf	5
Awassi	8
Chios	6
Friesian	6
Lacaune	26
Santa Ines	21
Sarda	5
Texel	6
Tsigai	9
Valachian	10

**Table 3:** Prevalent pathogens per number of studies.

Prevalent Pathogen	Number of Studies
CoNS	95
*S. aureus*	11
*E. coli*	3
*Bacillus* spp.	2
CoPS	1
Enterococci	1
*Mycoplasma* spp.	1
NaS	1
*S. hyicus*	1
Staphylococci	1
Mixed infections	1

NaS: Non-aureus Staphylococci.

**Table 4:** Species of CoNS in the selected studies.

Identified CoNS Species	Number of Total Isolations	Number of Respective Studies	% of Total Identified CoNS
*S. arlettae*	45	5	0.86
*S. auricularis*	199	15	3.82
*S. capitis*	50	16	0.96
*S. caprae*	249	25	4.78
*S. carnosus*	5	2	0.10
*S. caseolyticus*	3	1	0.06
*S. chromogenes*	782	44	15.00
*S. cohnii*	29	12	0.56
*S. condimenti*	1	1	0.02
*S. devriesei*	8	4	0.15
*S. epidermidis*	2046	61	39.25
*S. equorum*	145	14	2.78
*S. haemolyticus*	167	26	3.20
*S. gallinarum*	6	2	0.12
*S. hominis*	21	5	0.40
*S. jettensis*	1	1	0.02
*S. kloosii*	11	3	0.21
*S. lentus*	79	16	1.52
*S. lugdunensis*	19	5	0.36
*S. microti*	8	2	0.15
*S. muscae*	5	4	0.10
*S. pasteuri*	5	2	0.10
*S. petrasii*	3	1	0.06
*S. piscifermentans*	11	1	0.21
*S. rostri*	1	1	0.02
*S. saccharolyticus*	2	2	0.04
*S. saprophyticus*	33	12	0.63
*S. sciuri*	74	12	1.42
*S. simulans*	497	42	9.53
*S. vitulinus*	7	5	0.13
*S. warneri*	121	26	2.32
*S. xylosus*	361	40	6.92
*S. hyicus* ^1^	64	5	1.23
*S. intermedius* ^1^	21	4	0.40
*S. schleiferi* ^1^	134	9	2.57
Total	5213	77	100%

^1^ Species of Staphylococci that are, usually or regularly, coagulase positive. However, in a number of studies, these species are included in the group of CoNS isolates; therefore, they are listed in this table.

**Table 5:** Antibiotic resistance rates of the CoNS isolates in the included studies.

Class	Antibiotic ^1^	Resistant (R) Isolates/Tested CoNS per Study	Total R/ Total CoNS	Resistance Rate
Aminoglycosides	Gentamicin	0/7, 1/45, 6/78, 0/9, 0/7, 2/131, 0/1, 2/56, 0/24, 9/106, 5/75, 0/70, 0/11, 1/11, 1/112, 1/72, 0/4, 3/73, 0/20, 2/17, 0/8	33/937	3.52%
	Streptomycin	0/7, 17/56, 54/106, 4/11, 2/22, 2/24, 2/108, 0/4, 4/137	85/475	17.89%
	Kanamycin	0/7, 2/131, 3/56, 6/24, 2/73, 4/8	17/299	5.69%
	Neomycin	15/45, 0/7, 4/56, 0/11, 6/11, 1/24, 7/108, 36/99, 0/4, 8/137, 0/20	77/522	14.75%
Ansamycins	Rifampicin	0/1, 0/46, 0/24, 12/106, 1/112, 1/73	14/362	3.87%
Β-lactams	Amoxicillin	40/45, 0/7, 63/106, 10/24, 49/72, 1/108, 0/137, 0/17, 5/8	168/524	32.06%
	Amoxicillin–clavulanic acid	0/20, 2/78, 0/1, 1/39, 0/70, 0/11, 11/11, 15/99	29/329	8.81%
	Ampicillin	1/7, 33/78, 56/131, 13/56, 3/24, 20/39, 30/70, 2/11, 11/11, 1/11, 10/30, 13/24, 31/72, 4/108, 21/115, 29/73, 0/4, 6/137, 0/20, 11/33	295/1054	27.99%
	Cephalothin	0/7, 2/56, 0/70, 0/11, 0/30, 0/73	2/247	0.81%
	Cefoxitin	0/7, 0/1, 0/56, 2/72, 0/108, 3/115, 0/73, 1/137, 1/33	7/602	1.16%
	Oxacillin	0/7, 2/131, 0/1, 7/56, 0/24, 19/106, 16/75, 0/70, 1/11, 1/30, 7/72, 3/108, 0/4, 1/137, 0/17, 1/6	58/855	6.78%
	Penicillin	1/7, 40/45, 8/73, 9/9, 44/78, 56/131, 1/1, 14/46, 20/56, 5/24, 22/39, 23/75, 30/70, 3/11, 11/11, 8/11, 9/30, 23/24, 19/112, 29/72, 7/108, 29/115, 0/4, 7/137, 11/33, 7/8	436/1330	32.78%
Fluoro- quinolones	Ciprofloxacin	0/7, 0/1, 8/106, 0/39, 1/75, 0/24, 1/112, 0/4, 0/17, 0/8	10/393	2.54%
	Enrofloxacin	9/9, 4/78, 0/7, 1/46, 6/106, 0/70, 0/11, 3/99, 0/4, 0/73, 0/17, 0/8	23/528	4.36%
Glycopeptides	Vancomycin	0/1, 0/46, 7/24, 0/112, 2/72	9/255	3.53%
Lincosamides	Clindamycin	0/1, 0/39, 6/75, 14/115, 6/73, 1/33, 0/14, 0/4	27/354	7.63%
Macrolides	Erythromycin	8/78, 1/131, 0/1, 3/46, 5/56, 17/24, 46/106, 0/39, 3/75, 4/70, 2/11, 5/11, 2/22, 1/30, 3/112, 9/108, 10/115, 7/137, 2/33	128/1205	11.28%
Phenicols	Chloramphenicol	0/7, 0/1, 0/24, 0/75, 0/24, 2/73, 0/17, 0/6	2/227	0.88%
Pholate pathway inhibitor	Sulphamethoxazole–Trimethoprim	2/78, 0/46, 6/56, 1/115, 0/99, 0/4, 0/73, 0/33, 2/8	11/512	2.15%
Tetracyclines	Tetracycline	1/7, 0/7, 33/45, 9/9, 19/78, 13/131, 0/1, 4/46, 9/56, 48/106, 1/39, 2/75, 8/70, 3/11, 1/11, 17/30, 3/24, 12/112, 12/72, 1/108, 24/115, 27/99, 0/4, 8/73, 0/137, 16/33, 0/4	271/1503	18.03%

^1^ Only agents with ≥5 references and ≥200 tests are included in this table.

**Table 6:** Resistance rates in reference to the origin of the studies. In bold are the statistically significant results (when *p*< 0.005).

Antibiotic ^1^	Resistant Isolates/Tested CoNS per Continent (%)				
	Europe	South America	Asia	Total Rate	Chi-Square
Ampicillin	141/632 (22.31%)	76/232 (32.76%)	78/190 (41.05%)	27.99%	***p* < 0.00001**
Cefoxitin	5/467 (1.07%)	2/135 (1.48%)	ne	1.16%	*p* = 0.694939.
Gentamicin	7/263 (2.67%)	18/463 (3.89%)	7/166 (4.21%)	3.52%	*p* = 0.617974
Enrofloxacin	12/206 (5.83%)	6/128 (4.69%)	5/194 (2.58%)	4.36%	*p* = 0.276063
Erythromycin	51/560 (9.10%)	60/429 (13.99%)	15/216 (6.94%)	11.28%	***p* = 0.008058**
Oxacillin	7/424 (1.65%)	51/361 (14.13%)	ne	6.78%	***p* < 0.00001**
Penicillin	151/650 (23.23%)	125/399 (31.33%)	120/236 (50.85%)	32.78%	***p* < 0.00001**
Tetracycline	98/717 (13.69%)	104/505 (20.59%)	36/236 (15.25%)	18.03%	***p* = 0.004888**

^1^ Only agents with data from ≥10 studies and ≥500 isolates were included. ne: Not evaluated. Respective data were evaluated only when ≥3 studies and ≥100 isolates tested per region were available.

**Table 7:** Phenotypic assays for biofilm formation, detection of biofilm, and toxin-related genes.

Phenotypic Test for Biofilm Production	Detection of Biofilm-Associated Genes in CoNS	Detection of Toxin-Associated Genes in CoNS	Reference
1/39 (CRA)	13/39 (*ica*D)	---	[39]
1/36 (CRA)	2/36 (*ica*D)	---	[27]
33/53 (PA) ^2^	10/112 (*ica*ABCD), 16/112 (*bap*), 3/112 (*bhp*)	56/112 *(sea),* 19/112 *(seb)*, 15/112 *(sec),* 1/112 *(tsst)*	[26]
---	4/127 (icaA), 37/127 (*ica*C), 53/127 (*ica*D), 3/127 (*bap*), 0/57 (*bhp*)	35/127 *(sec)*, 0/127 *(sea*, *seb*, *sed, tsst)*	[25]
---	---	6/13 ^4^ (*sea*, *seb*), 0/13 (*sec-see*, *tsst*)	[21]
194/620 (CRA and PA)	43/116 (*ica*A), 28/116 (*ica*C), 30/116 (*ica*D), 40/116 (*bap*), 58/116 (*eno*), 20/116 (*clfa*)	*---*	[55]
149/222 (CRA and PA)	---	---	[57]
---	---	0/24 *(sea-see, seg-sel, seq, tsst)*	[63]
2/199 (CRA)	6/199 *(ica*A), 5/199 (*ica*D), 113/199 (*bhp*), 11/199 (*aap*), 199/199 *(embp*)	0/199 *(sea-see, tsst*)	[64]
0/73 (CRA)	15/73 (*ica*A), 20/73 (*ica*D), 0/73 (*bap*), 49/73 (*embp*), 45/73 (*eno, fbe*), 6/73 (*aap*)	---	[65]
41/125 (PA)	3/125 (*ica*A), 1/125 (*ica*D), 37/125 (*bhp*), 22/125 (*aap*), 63/125 (*embp*)	0/125 *(sea-see, tsst)*	[67]
8/109 (CRA), 26/327 (PA)	---	*---*	[140]
74/102 ^3^ (PA)	1/24 (*bap*), 2/24 (*ica*A), 15/24 (*ica*D)	---	[138]
---	---	1/102 (*sea, sec*), 2/102 (*seb, sed*), 0/102 (*see*)	[77]
8/44 (CRA)	---	*---*	[87]
---	---	13/40 (*sea-see, seg-sej*) ^1^, 0/40 *(eta, etb, tsst)* 2/40 *(plv)*	[106]
28/70 (CRA)	42/70 (*ica*A and *ica*D)	---	[103]
---	---	4/27 (*plv*)	[107]
Total ^5^			
CRA:48/570 (8.4%) PA:174/607 (28.7%)	*aap:* 39/397 (9.8%), *bap:* 60/452 (13.3%), *bhp:* 153/493 (31%), *embp*: 311/397 (78.3%), *ica*A: 115/734 (15.7%), *ica*D: 181/809 (22.4%)	*sea-see*, *seg-sel, seq* ^6^: 129/742 (17.4%) *tsst*: 1/640 (0.002%)	

CRA: Congo Red Agar. PA: Plate adhesion method. ^1^ All of these 13 CoNS isolates harbored at least one of the respective genes. ^2^ Only bacteria from cases of mastitis included. ^3^ Isolates of both caprine and ovine origin included. ^4^ Only isolates from subclinical mastitis. ^5^ Only in cases with available data from ≥3 relevant articles. ^6^ At least one of the respective genes.

**Table 8:** Procedures of microbiological examination per number of respective studies.

Microbiological Examination Procedure	Number of Respective Studies
Conventional phenotypic tests for identification ^1^	116
Inoculation in both non-selective and selective media MacConkey agar Mannitol salt agar Other selective media	71 47 23 25
Inoculation only in non-selective media ^2^	66
Commercial biochemical identification kit (total) API Staph API Strep Other API kits (20E, 20NE, coryne, etc.) Other biochemical identification kits ^3^	44 25 5 9 13
Molecular assays	37
MALDI-TOF MS	14
VITEK II	10
Susceptibility estimated through disc diffusion	39
Susceptibility estimated through MIC ^4^	16

^1^ For example, growth, colony morphology, hemolysis, GRAM staining, catalase, oxidase, coagulase-test, CAMP test, and other biochemical tests. ^2^ Initial inoculation of a quantity of each milk sample. ^3^ STAPHYtest (Erba-Lachema, Brno, Czech Republic), Crystal™ Identification Systems Gram-Positive ID kit (Becton, Dickinson and Company, Franklin Lakes, NJ, USA). ^4^ Including the 10 studies where VITEK II was used.

**Table 9:** Generic information of the studies included in this review.

Country/Region	Breed	Total Milk Samples Tested	Total Number of Isolated CoNS	Prevalent Isolated Pathogen	Reference
Algeria/Oran, Mascara, Relizane	Ouled-Djellal	105	15	Enterococci	[121]
Algeria/eastern	Ouled-Djellal	214	34	CoNS	[122]
Austria	na	267	75	CoNS	[123]
Austria	na	908	130	CoNS	[124]
Brazil/Pernambuco	Santa Ines	244	80.2% *	CoNS	[9]
Brazil	Santa Ines	na	79.2% *	CoNS	[10]
Brazil/Sao Paolo	Santa Ines	48	23	CoNS	[11]
Brazil/Bauru	Santa Ines	309	85	CoNS	[12]
Brazil/Para	Santa Ines	352	7	CoNS	[13]
Brazil/Sao Paolo	Santa Ines	125	18	CoNS	[14]
Brazil/Santa Catarina	na	164	25	NaS	[38]
Brazil/northeast	Santa Ines	340	56	CoNS	[15]
Brazil/Montes Claros	Santa Ines	286	64	CoNS	[16]
Brazil/Pernambuco, Bahia	na	na	106	na	[39]
Brazil/Pernambuco	na	na	39	CPS	[40]
Brazil/Capao do Leao	Corriedale, Texel	176	15	CoNS	[34]
Brazil/Sao Paolo	Santa Ines	448	75	CoNS	[17]
Brazil/Chapeco SC	Lacaune-Ile de France, Lacaune-Texel	na	11	CoNS	[27]
Brazil/Sergipe State	Santa Ines	330	85	CoNS	[18]
Brazil/Sao Carlos	variable	911	92	CoNS	[35]
Brazil/Sao Carlos	Santa Ines, Morada Nova	393	39	CoNS	[22]
Brazil/Sao Carlos	Santa Ines	1081	122	CoNS	[19]
Brazil/Sao Carlos	variable	1457	118	CoNS	[36]
Brazil/Rio Grande do Sul	Lacaune	315	55	CoNS	[28]
Brazil/Sao Carlos	Santa Ines, Morada Nova	584	57	CoNS	[23]
Brazil/Sao Paolo	Santa Ines, Morada Nova	907	134	CoNS	[24]
Brazil/Rio Grande do Sul	Lacaune	71	39	CoNS	[29]
Brazil/Sao Paolo	Santa Ines, Bergamacia	484	53	na	[26]
Brazil/Santa Catarina	Lacaune	179	22	CoNS	[30]
Brazil/Santa Catarina	Lacaune	492	68	CoNS	[31]
Brazil/northeast	na	na	72	na	[41]
Brazil/Sao Paolo	variable	1457	123	CoNS	[37]
Brazil/Sao Paolo	Santa Ines, Morada Nova	na	57	na	[25]
Brazil/Parana State	Santa Ines	256	36	CoNS	[20]
Brazil/Chapeco SC	Lacaune	30	4	*S. hyicus*	[32]
Brazil	Santa Ines	532	68	CoNS	[21]
Brazil/Minas Gerais	Lacaune	109	41	CoNS	[33]
Bulgaria	Lacaune	30	17	CoNS	[125]
Bulgaria	variable	120	8	CoNS	[126]
Czech Republic	Tsigai	60	1	*S. aureus*	[127]
Czech Republic/Slovakia	Tsigai	89	3	*S. aureus*	[128]
Egypt/Fayoum Governorate	Balady	196	38	CoNS	[96]
Egypt/Kafr El Seikh Governorate	Native breeds	245	66	CoNS	[97]
Egypt/Assiut Governorate	na	198	9	CoNS	[98]
Egypt/El-Fayoum, Beni-Suef, Giza	na	189	8	*S. aureus*	[100]
Egypt/Sharkia Governorate	na	216	9	Mixed infections	[99]
Egypt	na	455	18	*S. aureus*	[101]
Ethiopia/Kafta Humera	Begayd, Abergelle	135	11	CoNS	[112]
Ethiopia/Haramaya	Native breeds	24	2	Staphylococci	[113]
Ethiopia/Jimma	Native breeds	372	19	*S. epidermidis*	[114]
France/southwest	na	3758	na	CoNS	[129]
France/Roquefort	Lacaune	1122	325	CoNS	[130]
Germany/middle, northern	East Friesian, Lacaune, mixbreeds	328	24	CoNS	[131]
Greece	Karagouniko	480	2	CoNS	[42]
Greece	Karagouniko	924	11	CoNS	[43]
Greece	Chios	916	73	CoNS	[44]
Greece	na	206	41	CoNS	[50]
Greece	Mytilene	461	91	CoNS	[51]
Greece/Giannitsa	Chios	94	11	CoNS	[45]
Greece/central Greece	Lacaune cross	186	13	CoNS	[47]
Greece/central Greece	Lacaune	na	7	CoNS	[48]
Greece/throughout country	na	na	90	CoNS	[52]
Greece/throughout country	variable	2198	454	CoNS	[53,54,55,56]
Greece/Thessaly	variable	3637	272	CoNS	[57]
Greece/southern	na	240	44	CoNS	[49]
Greece/Crete	Sfakia	9624	652	CoNS	[58]
Greece/throughout country	variable	na	33	na	[59]
Greece/Central Macedonia	Chios	na	60	CoNS	[46]
Hungary/eastern	na	62	4	*S. aureus*	[132]
Iran/Urmia Province	na	209	44	CoNS	[88]
Iran/Shahrekord Region	Native breeds	400	7	*Mycoplasma* spp.	[89]
Iran/Tabriz	na	260	18	CoNS	[91]
Iran/Nagadeh	Ghezel	146	5	*E. coli*	[92]
Iran/Shahrekord Region	Native breeds	600	22	*S. aureus*	[90]
Iran/Semnan Region	na	1192	87	CoNS	[93]
Iran/West Azerbaijan Province	Makui, Ghezel, crossbreds	900	24	na	[95]
Iran/Semnan Province	Sangsari	196	50	CoNS	[94]
Israel	Israeli-Assaf	na	36	na	[115]
Israel	Israeli-Assaf	318	134	CoNS	[116]
Israel	Assaf	61	29	na	[117]
Italy/Sardinia	na	42	10	CoNS	[68]
Italy/Sardinia	Sarda	2198	61	CoNS	[60]
Italy/Sicily	Valle del Belice	8843	2316	CoNS	[71]
Italy/Sardinia	Sarda	2201	226	na	[61,62]
Italy/Sardinia	Sarda	2828	820	CoNS	[69]
Italy/Sardinia	Sarda	na	24	CoNS	[63]
Italy/Sardinia, Lazio, Sicily, Tuscany	na	23,040	4162	CoNS	[73]
Italy	Valle del Belice	20,519	7951	CoNS	[72]
Italy/Sardinia, Sicily	Sarda, Valle del Belice, Comisana	123	75	CoNS	[74]
Italy/Piedmont	Variable	41	32	CoNS	[75]
Italy/Sardinia	Sarda	1498	134	CoNS	[70]
Italy/Sardinia	na	na	199	na	[64]
Italy/Tuscany, Lazio	na	120	73	na	[65]
Italy/Sardinia	na	na	190	na	[66]
Italy/Sardinia	na	na	124	na	[67]
Jordan/Southern	Awassi	1147	10	*S. aureus*	[118]
Jordan/Al Balqa	Awassi	220	11	*S. aureus*	[120]
Jordan/Northern	Awassi	279	38	CoNS	[119]
Mexico/Pajacuaran	Rambouillet, Criolla, Friesian	150	11	CoNS	[133]
Nigeria/Bauchi State	na	108	2	*E. coli*, *S. aureus*	[134]
Palestine	Awassi	40	12	CoNS	[135]
Poland/Zelazna	Native breeds	634	108	Staphylococci	[136]
Portugal/Evora, Alentejo	Variable	414	249	CoNS	[139]
Portugal/Alentejo	na	na	109	na	[140]
Portugal-Brazil	na	138	20	*S. aureus*	[138]
Romania/western	Merinos of Transylvania, Turcana	30	1	na	[137]
Serbia	na	13,218	25	*E. coli*	[141]
Slovakia/eastern	Tsigai, Valachian, Tsigai-Merino	540	214	CoNS	[76]
Slovakia	Valaska	820	31	CoNS	[77]
Slovakia/eastern	Improved Valaska	3466	288	na	[79]
Slovakia/Gelnica, Trebisov	Improved Valaska, Tsigai, Lacaune	494	76	CoNS	[78]
Slovakia	Variable	310	99	CoNS	[81]
Slovakia	Tsigai	20	8	CoNS	[82]
Slovakia/northern	Valaska	40	9	CoNS	[83]
Slovakia	Improved Valaska, mixbreeds	3466	158	*S. aureus*	[80]
Slovakia	Tsigai, Lacaune, Improved Valachian	407	104	CoNS	[84]
Slovakia	Lacaune	98	31	CoNS	[85]
Slovakia/eastern and northern	Lacaune, improved Valachian, crossbreeds	981	na	CoNS	[86]
Slovakia/eastern	Improved Valachian, Tsigai, Lacaune	940	44	CoNS	[87]
Spain	Churra	2022	361	CoNS	[142]
Spain/Barcelona	Manchega, Lacaune	216	na	CoNS	[143]
The Netherlands	Texel	388	131	CoNS	[144]
The Netherlands	Texel	920	251	CoNS	[145]
Turkey/Hatay	Awassi	1458	75	CoNS	[102]
Turkey/Hatay	Awassi	na	50	na	[104]
Turkey/Kirikalle	na	1604	41	CoNS	[105]
Turkey/Kirikalle	na	na	40	na	[106]
Turkey/Hatay	Awassi	na	70	na	[103]
Turkey/Afyonkarahisar	Pirlak	464	31	na	[107]
UK/Midlothian	Blackface cross Border Leicester	492	33	CoNS	[146]
UK/Penicuik	Scottish Blackface cross Leicester	219	21	CoNS	[147]
USA/Wisconsin	Variable ^1^	214	73	CoNS	[108]
USA/Wyoming	Rambouillet	22	4	*Bacillus* spp.	[109]
USA/Montana, Idaho	Variable	243	25	*Bacillus* spp.	[110]
USA/Wyoming	na	174	na	CoNS	[111]

CoNS: Coagulase-negative Staphylococci. na: Not available. NaS: Non-aureus Staphylococci. ^1^ Variable: When >3 breeds are included in the study. * Only percentages were available in these studies.

## Data Availability

Data collected in this study is contained within the article.
